# NEIGHBORHOOD COHESION, NEIGHBORHOOD PHYSICAL DISADVANTAGE, AND END-OF-LIFE CARE: HOW NEIGHBORHOOD MATTERS

**DOI:** 10.1093/geroni/igad104.2368

**Published:** 2023-12-21

**Authors:** Kedong Ding, Yifan Lou

**Affiliations:** Case Western Reserve University, Cleveland, Ohio, United States; Yale University, New Haven, Connecticut, United States

## Abstract

**Background:**

Many studies have focused on the association between individual-level factors and end-of-life care. Yet neighborhood-level factors and end-of-life care are understudied and thus remain unclear. This study aims to examine the association between neighborhood characteristics and U.S. older adults’ end-of-life care to fulfill the existing research gaps. Method: Data were from the 2008 to 2016 Health and Retirement Study exit interview (n = 1,561) who answered left behind questionnaires. Participants included community-dwelling adults aged 55 years or older who died between waves. End-of-life care is measured from three dimensions: health service utilization, quality of life, and advance care planning; Neighborhood characteristics include neighborhood cohesion, which was assessed with 4 items to measure the extent to which respondents feel about the neighborhoods and neighbors’ trust; neighborhood physical disadvantage, which was assessed with 4 items to measure the extent to which respondents feel about the neighborhoods’ physical problems. Logistic regressions with sociodemographic and health controls were conducted.

**Results:**

A higher level of perceived neighborhood cohesion and lower level of perceived neighborhood physical disadvantages are significantly associated with a lower likelihood of being admitted to the ICU, getting life support, and having trouble with pain. Higher neighborhood cohesion is also associated with increased odds of having a living will (OR= 1.10, p < 0.05). Neighborhood characteristics are not associated with end-of-life hospitalization, use of hospice, depression, and having end-of-life care conversations.

**Conclusion:**

This study demonstrated how neighborhood characteristics will influence end-of-life care service utilization, quality of care, and advance care planning.

